# The Role of Cysteine Peptidases in Hematopoietic Stem Cell Differentiation and Modulation of Immune System Function

**DOI:** 10.3389/fimmu.2021.680279

**Published:** 2021-07-15

**Authors:** Milica Perišić Nanut, Urša Pečar Fonović, Tanja Jakoš, Janko Kos

**Affiliations:** ^1^ Department of Biotechnology, Jožef Stefan Institute, Ljubljana, Slovenia; ^2^ Faculty of Pharmacy, University of Ljubljana, Ljubljana, Slovenia

**Keywords:** cysteine cathepsins, cystatins, hematopoietic stem cell, immune cell development, extralysosomal activity

## Abstract

Cysteine cathepsins are primarily involved in the degradation and recycling of proteins in endo-lysosomal compartments but are also gaining recognition as pivotal proteolytic contributors to various immune functions. Through their extracellular proteolytic activities within the hematopoietic stem cell niche, they are involved in progenitor cell mobilization and differentiation. Cysteine cathepsins, such as cathepsins L and S contribute to antigen-induced adaptive immunity through major histocompatibility complex class II antigen presentation whereas cathepsin X regulates T-cell migration. By regulating toll-like receptor signaling and cytokine secretion cysteine cathepsins activate innate immune cells and affect their functional differentiation. Cathepsins C and H are expressed in cytotoxic T lymphocytes and natural killer cells and are involved in processing of pro-granzymes into proteolytically active forms. Cytoplasmic activities of cathepsins B and L contribute to the maintenance of homeostasis of the adaptive immune response by regulating cell death of T and B lymphocytes. The expression pattern, localization, and activity of cysteine cathepsins is tightly connected to their function in immune cells. Furthermore, cysteine cathepsins together with their endogenous inhibitors, serve as mediators in the interplay between cancer and immune cells that results in immune cell anergy. The aim of the present article is to review the mechanisms of dysregulation of cysteine cathepsins and their inhibitors in relation to immune dysfunction to address new possibilities for regulation of their function.

## Introduction

Lysosomes are single-membrane degradative organelles that are essential for cell homeostasis. Through proteolytic processing and degradation, lysosomes are involved in numerous cellular processes. Cathepsins constitute a subset of lysosomal peptidases and, according to their catalytic type, are classified as serine (A and G), aspartic (D and E; pepsin family A1A), or cysteine (B, C, L, F, H, K, O, S, V, X, and W; papain family C1A) cathepsin peptidases (1). Cysteine cathepsins gained much attention in the past decades due to the critical role they play in cellular compartments other than those of the endo-lysosomal system and within the extracellular milieu (2). Apart from their important roles in the immune response, such as antigen processing and presentation as well as processing and activating various proteins and hormones, cysteine cathepsins also play important roles in immune cell development, regulation of the immune response and immune homeostasis, and aging of the immune system.

The majority of cysteine cathepsins are ubiquitous; however, the cellular expression patterns of some are strictly linked to their biological function (3, 4). Cathepsin K (CatK) is abundant in osteoblasts, osteoclasts, macrophages, synovial fibroblasts, and epithelial cells (5), cathepsin V (CatV) expression is restricted to the thymus and testis (6), and cathepsin S (CatS) is expressed in antigen-presenting cells (APCs) such as B cells and dendritic cells (DCs) (7). The highest expression of cathepsin X (CatX) is detected in immune cells of myeloid lineage such as macrophages, DCs, and microglia; however, its expression was also detected in B lymphocytes and natural killer (NK) cells (8, 9). Cathepsin W is limited to cytotoxic lymphocytes, with significantly higher expression levels in NK cells than those in cytotoxic T lymphocytes (CTLs) (10).

As peptidases catalyze the irreversible cleavage of peptide bonds, any dysregulation in peptidase expression, localization, or proteolytic activity can disrupt cellular homeostasis. Their activity is regulated through the regulation of gene expression, post-translational modifications, activation of zymogens, the accessibility of the susceptible peptide bond in the substrate, their compartmentalization, and their endogenous inhibitors (11, 12). Dysregulated cathepsin activity was shown to be the contributing factor in diseases such as bronchial asthma, atherosclerosis, rheumatoid arthritis, osteoarthritis, and cancer (13).

In this review, we focus primarily on lysosomal and extra-lysosomal cysteine cathepsins and their role in the development of the immune system and in the regulation of the immune response. A comprehensive review of the role of cysteine cathepsins in myeloid cell differentiation and function was published by our group recently (14) therefore our aim is to highlight the recent developments in the field and emphasize the importance of cysteine peptidases and their endogenous and exogenous inhibitors for normal development, function and homeostasis of the lymphoid cells of the immune system.

## The Structure and Localization of Cysteine Cathepsins

### Structure

Due to their structural similarity to the plant cysteine peptidase papain, isolated from *Carica papaya* in 1937, cysteine cathepsins were designated as the C1 family, clan CA of the MEROPS peptidase classification system (15, 16). Papain-like peptidases are composed of the N-terminal left domain, which contains three α helices, and the C-terminal right domain, which forms the β-barrel fold. At the interface of both domains lies the V-shaped active site cleft that includes the conserved catalytic pair Cys-His. Cathepsins are monomeric proteins with an average molecular weight of 20–35 kDa in their mature form; the only exception is cathepsin C (CatC; also named dipeptidylpeptidase I or DPPI) that exists as a 200 kDa homotetramer (17–19).

Cathepsins are synthesized as precursor preproenzymes and are shuttled to the endoplasmic reticulum, where their N-signaling sequence is cleaved, and the protein is N-glycosylated. High-mannose glycans are essential for binding to mannose 6-phosphate receptors, packaging into clathrin-coated vesicles, and sorting to lysosomes. In addition, lysosomal hydrolases can be sorted in mannose 6-phosphate receptor-independent pathways (20, 21). The active site of cathepsin zymogens is blocked by the N-terminal proregion, which acts as a reversible inhibitor to prevent premature enzyme activation while also containing sorting signals and assisting in correct folding. Conversion to the mature form takes place in the acidic and reducing milieu of lysosomes, in which the propeptide is cleaved either *via* autocatalytic processing or trans-activation by another peptidase (22). In an additional proteolytic step, the cathepsins B, H, and L can be processed from a single chain to disulfide-linked heavy and light chains, without the loss of their activity (23). With few exceptions, cathepsins are endopeptidases. Exopeptidases exhibit additional structural features that restrict access to the active site and form electrostatic bonds with the C- or N-termini of substrates (24).

Cathepsins have relatively small substrate binding area, which encompasses the S1, S2, and S1’ binding pockets. The remaining active-site subsites (S3 and S2’) are less structurally defined and do not depend on main chain interactions with the substrate. Substrate binding mainly depends on the characteristics of the P2 substrate residue (25, 26) and several studies have demonstrated a strong preference of cysteine cathepsins for small hydrophobic amino acid residues (Leu, Val, Ile) in the P2 position of the substrate (27). Although cathepsins have broad and partially overlapping substrate specificities there are several examples of higher substrate specificity. For example, β2 cytoplasmic tail of integrin (28, 29) and profilin 1 were validated as a CatX targets (30), whereas CatH, as a monoaminopeptidase, processes talin at its N-terminal head domain that contains the integrin binding site (31). There are also examples of substrates like collagen, osteocalcin, cytokines, and chemokines, which were only cleaved by a subset of cathepsins (27, 32).

### Localization

**Figure 1:**
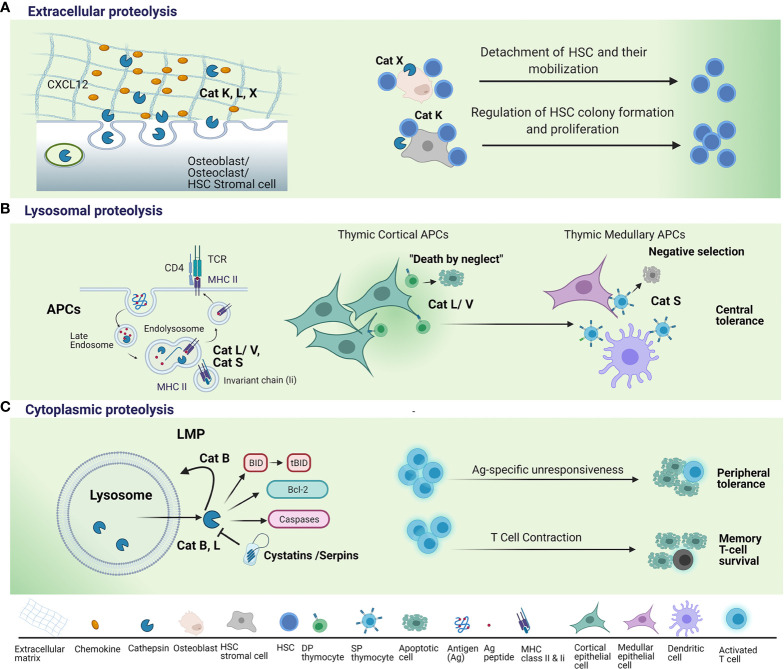
Regulation of T lymphocyte survival by cysteine cathepsins. **(A)** When secreted into the extracellular space, cysteine cathepsins, such as cathepsin K (Cat K), cathepsin L (Cat L) and cathepsin X (Cat X) remodel extracellular matrix (ECM) and degrade chemokines, such as C-X-C motif chemokine 12 (CXCL12), necessary for hematopoietic stem cells (HSCs) proliferation and their retention within the HSC niche. This leads to detachment of HSCs and their mobilization from the HSC niche. Lack of Cat K within the niche microenvironment results in reduced proliferation of HSC and their cell colony formation and proliferation. **(B)** Endolysosomal cysteine cathepsins are essential for two steps in major histocompatibility complex (MHC) class II antigen presentation: (a) the degradation of invariant chain (Ii) to class II-associated invariant chain peptide (CLIP) to permit dissociation of CLIP from class II molecules and subsequent peptide binding; and (b) the generation of antigenic peptide fragments from larger polypeptide/protein moieties. Cat L, in mouse cortical thymic antigen presenting cells (APC) and cathepsin V (Cat V) in human APC, have non-redundant roles in degradation of invariant chain (Ii) to class II-associated invariant chain peptide (CLIP). Cathepsin S (Cat S) is involved in MHC class II antigen presentation in medullary thymic APCs. Thymocyte, whose T-cell receptor (TCR) fails to recognize an antigenic peptide/MHC complex on cortical APCs, fails to receive a survival signal i.e. death by neglect. Thymocytes, whose TCR strongly binds to antigenic peptide/MHC complex on medullary APCs are eliminated i.e. negative selection. **(C)** Following the induction of apoptosis, lysosomal cathepsins are released from lysosomes through lysosomal membrane permeabilization (LMP). Cathepsins B (Cat B) and L perform their pro-apoptotic function in the cytosol by cleaving Bid, a pro-apoptotic Bcl-2 family member, anti-apoptotic Bcl-2 family protein or cleaving and directly activating caspases. Cysteine peptidase inhibitors, such as serpins and cystatins, may regulate cathepsin activity in the cytosol. Cat B further promotes LMP and the release of lysosomal constituents. Cathepsins B and L released through LMP have been implicated in apoptosis triggered by supraoptimal activation of T lymphocytes i.e. high dose tolerance. Cat B pro-apoptotic actions in the cytosol have been shown to mediate apoptosis induction upon critical phase of immunity whereby the vast majority of effector T cells die by apoptosis i.e. T cell contraction. Upregulation of endogenous inhibitor (Spi) 2A enables survival of memory T cells; antigen (Ag).

The endo-lysosomal internal acidic pH favors cathepsin activity and induces conformational changes in the substrates, leading to their proteolytic cleavage by cathepsins (33). However, cysteine cathepsins can be found in cellular compartments other than endo-lysosomes and also in the extracellular space (27) (**Figure 1**). Kinetic studies *in vitro* showed that irreversible unfolding occurs at neutral pH for all cathepsins (34), except for the more stable CatS (35). However, binding to their substrates or endogenous inhibitors (36) as well as allosteric interactions with glycosaminoglycans (GAGs) and other negatively charged molecules (37) stabilize cysteine cathepsins and preserve their activity even at higher pH outside endo-lysosomes. Several studies showed interdependence between cysteine cathepsins and GAGs [reviewed in (37)]. Cysteine cathepsins are capable of cleaving proteoglycan core proteins and thereby release GAGs; in turn GAGs affect both the activity and stability of cysteine cathepsins in the extracellular space (37). In addition, acidification of the cytosol observed under certain conditions such as apoptosis enables cathepsins to mediate key physiological processes (38, 39).

Cells of hematopoietic origin, such as platelets, neutrophils, eosinophils, mast cells, macrophages, and cytotoxic cells, contain a specialized lysosomal compartment that can be secreted into the extracellular environment in response to specific stimuli (27, 40, 41). These “secretory lysosomes” include cytotoxic granules, major histocompatibility (MHC) class II compartments, and basophil and azurophil granules (42). Like conventional lysosomes, secretory lysosomes contain degradative proteins and similar internal acidic pH but are distinguished by their ability to undergo controlled secretion (43). In this fashion, multiple aspects of the immune response are regulated through extracellular proteolysis by cysteine cathepsins. For example, CatX translocation from lysosomes to the perimembrane region facilitates migration of immune cells and depends on the interaction of CatX with the cytoplasmic tails of β_2_ integrin receptors (28, 44). After vesicular exocytosis, cathepsins can be delivered to the cell membrane, where they associate with components of the extracellular matrix (ECM) or remain adhered to the membrane, as was shown for cathepsins B, S, and X. CatB localizes to the cell membrane by binding to surface annexin II (45, 46), whereas cathepsins S and X interact with α_ν_β_3_ integrins (47, 48). Likewise, osteoclasts reorganize their lysosomal compartment by translocating lysosomal glycoproteins and proton pumps to the ruffled border membrane to secrete lysosomal enzymes at the site of bone resorption (49).

Apart from vesicular exocytosis, different stimuli promote lysosomal release of cathepsins and their translocation to other cellular compartments (27). Namely, cathepsins have also been detected in cell nuclei, in which they are involved in the regulation of gene expression and the cell cycle (50). Cathepsins without nuclear localization motifs rely on chaperone molecules (such as Snail and HLA B-associated transcript 3) for translocation, which are often enhanced in cancer cells (51, 52). In the nucleus, cathepsins degrade the regulators of nuclear DNA repair machinery, including BRCA1 [cathepsins B (53) and S (54)] and 53bp1 (cathepsin L) (55).

In the past two decades, it was shown that by regulating apoptosis in immune cells, cathepsins play important roles in the development, maturation, and function of lymphocytes. For their proapoptotic function, cathepsins must be released from lysosomes into the cytosol by lysosomal membrane permeabilization (LMP) (56) (**Figure 1**). Unlike lysosomal rupture, which results in necrosis, LPM results in a slow release of cathepsins and the selective cleavage of a few targets. LMP is induced by a plethora of stimuli including oxidative stress, lysosomotropic agents, and certain endogenous cell death receptors. The cytosolic cathepsins B, D, and L indirectly support activation of caspase-dependent apoptosis by promoting mitochondrial membrane destabilization through cleavage of the pro-apoptotic BH3-only protein, Bid, and degradation of the B-cell lymphoma-2 protein, Bcl-2 (57, 58). Simultaneously, cathepsins are involved in the degradation of the anti-apoptotic proteins Bcl-2, Bcl-xL, Mcl-1, and XIAP (X-linked inhibitor of apoptosis), thus promoting apoptosis (59). Furthermore, CatB amplifies the feedback loop by potentiating the extent of LMP (60). Cathepsins also modulate other cell death pathways, mostly detected in immune cells, including programmed necrosis, i.e., necroptosis. Necroptosis is initiated by various stimuli and requires the kinase activity of receptor-interacting serine/threonine kinase 1. In macrophages, cathepsins B and S cleave receptor-interacting protein kinase 1 and thus limit necroptosis (61). CatB is responsible for the proinflammatory response during lytic programmed cell death, i.e., pyroptosis, as it activates the NLR family pyrin domain containing 3 (NLRP3) NLRP3 inflammasome and caspase-1 that catalyze the production of the interleukins (IL) IL-1β and IL-18 (62).

## Cysteine Cathepsins in Immune Cell Development and Differentiation

Hematopoietic stem cells (HSCs) are retained and regulated in specialized perivascular niches consisting of various support cells such as endothelial cells, osteoblasts, macrophages, megakaryocytes, perivascular cells, and specialized reticular cells (63). The complex microenvironment of the HSC niche enables HSC maintenance, proliferation, and differentiation into mature blood and immune cells (63). During embryogenesis in both mice and humans, hematopoiesis occurs in multiple waves throughout the developing embryo and fetus, including the aorta-gonad-mesonephros, fetal liver, and placenta before HSC eventually homing to the bone marrow (BM) (64, 65).

### Lymphoid Organs

While there are multiple studies implicating cysteine cathepsins in the regulation of bone ECM homeostasis (2, 66), very little is known about their involvement in hematopoiesis and the maintenance of the HSC niche ECM. The ECM of the HSC niche contains structural collagens, fibronectin, laminin, and proteoglycans and is a rich reservoir of local growth factors, cytokines, and other bioactive molecules (63). Multiple components of this niche ECM are similar to the components of bone ECM and are subject to regulation by the same peptidases and their natural inhibitors (**Table 1**). For example, CatK is known to degrade type I collagen that is critical for osteoclast bone resorptive activity (85); however, type I collagen was also found to be required for the optimal survival of HSCs (86). Furthermore, various cysteine cathepsins were shown to cleave the protein core of proteoglycan, a major constituent of the ECM. Proteoglycans such as heparan sulfate are implicated as critical components of the HSC niche (87), and the inhibition of heparan sulfate proteoglycan production by BM osteolineage stromal cells results in HSC egress from the BM niche into the peripheral circulation (67). Another protein that is tightly involved in the regulation of ECM homeostasis and is a substrate of cathepsins is heparanase, an endo-β-D-glucuronidase that degrades GAG-heparan sulfate in the ECM. Cathepsin L (CatL) is essential for the activation of heparanase (88). Several other ECM substrates of CatK, such as stem cell factor, osteopontin, and the chemokine SDF-1 (also called CXCL12) all have documented effects on HSCs and important for HSC maintenance (89, 90).

**Table 1:** The role of cysteine cathepsins in immune cell development and homeostasis.

Immune cell development	
***Bone marrow***	Osteoblast (67) secreted Cathepsin K degrades type I collagen (60), osteopontin, and CXCL12 (65). Cathepsins B, L, and X digest CXCL12 *in vitro* (67). Cathepsin L is involved in activation of heparanase (64).
	Cathepsin K is required for osteoclast engagement in response to the microenvironmental stimuli (59).
	Cathepsin K derived from HSC supportive stromal cells (68, 69) affects LSK cell colony formation and proliferation *in vitro* and their repopulating activity and maintenance of lymphopoiesis *in vivo* (70).
	*In vitro*, TLR9 signaling promotes caspase-independent cell death via a cathepsin B-dependent mechanism in pro-B cells (71).
***Thymus***	Cathepsin L is critical MHC class II-mediated peptide presentation, acting both in Ii degradation (72) and in the generation of MHC class II-bound peptide ligands presented by thymic cortical APCs in mice (73).
	Cathepsin V expressed in thymic cortical APCs in human thymus, participates in Ii processing (74)
	Cathepsin S in human thymic DCs, is involved in negative selection/elimination of autoreactive T cells (75).
	Cathepsin L is critical for CD1d presentation to NKT cells *in vitro* and their selection *in vivo* (76).
	Cathepsin L is critical for maturation of NKT cells (77).
***Immune cell homeostasis***	
	Cathepsins B and L activities are essential for T lymphocyte apoptosis in response to high concentration of antigen *in vitro* (78, 79).
	Cathepsin B pro-apoptotic activity limits the long-term maintenance of memory CD8+ T cell populations (80).
	Cathepsins B and L are essential for antigen-dependent germinal center B cell death (81).
	Cathepsin B was identified as a negative feedback regulator of lysosomal biogenesis and autophagy (82).
	Cathepsin H, trough cleavage of talin (30) could be involved in maintaining the homeostasis and survival of the regulatory T (Treg) cell pool (83).
	Pro- and mature forms of cathepsin X interact with β2 integrin receptors, thus modulating their affinity for extracellular ligands (84).

CXCL12, C-X-C motif chemokine 12; HSC, Hematopoietic stem cells; NKT, Natural killer T cells; TLR9, Toll-like receptor 9; MHC, major histocompatibility complex molecules; Ii, invariantchain; APC, antigen presenting cell; LSK Lineage^-^Sca^+^Kit^+^cells; dendritic cells; (DCs), dendritic cells.

**Figure 2:**
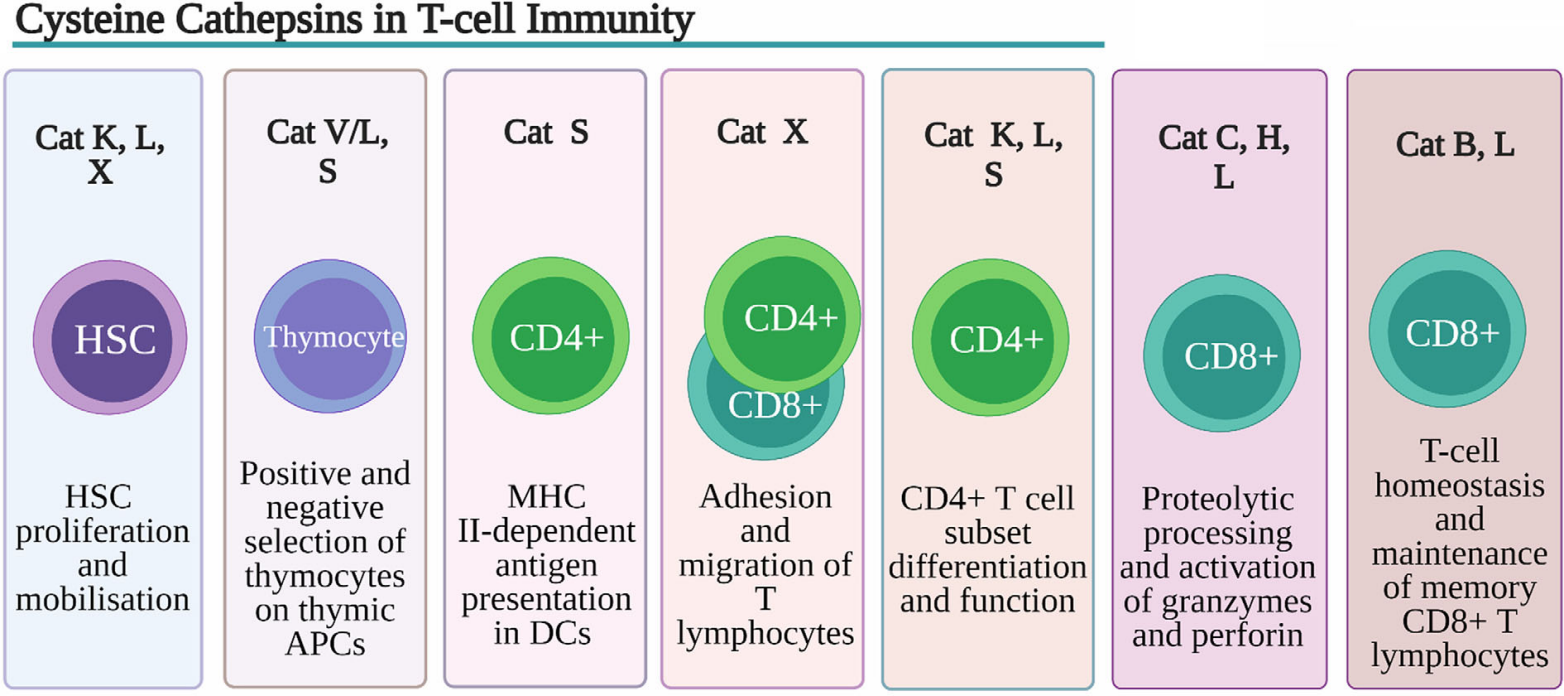
Role of Cysteine Cathepsins in T-cell immunity. Despite their overlapping substrate specificities several cysteine peptidases have been shown to play non-redundant roles in development, function and homeostasis of T cells. Cathepsin K (Cat K), cathepsin L (Cat L) and cathepsin X (Cat X) are involved in remodeling of extracellular matrix (ECM) and degradation of chemokines, necessary for hematopoietic stem cells (HSCs) proliferation and retention within the HSC niche. Cat L, in mouse cortical thymic antigen presenting cells (APC) and cathepsin V (CatV) in human APC, are indispensable for degradation of invariant chain (Ii) to class II-associated invariant chain peptide (CLIP) necessary for the process of positive thymic selection. In human professional APCs cathepsin S (Cat S) was shown to be indispensable for MHC class II antigen presentation. By regulating toll-like receptor (TLR) 9 and TLR7 signaling in dendritic cells (DC) CatK affects Th17 polarization. Cathepsin S (CatS), through activation DC protease-activated receptor-2 (PAR2) and upregulation of IL-6 secretion drives splenic DC-dependent Th17 differentiation. Cat K through cleavage of TLR7 blocks immuno-suppressive activity regulatory T cells (Tregs). Through processing of β2 integrin receptors Cat X modulates their affinity for extracellular ligands thus affecting the adhesion and migration of T lymphocytes. Cat C and cathepsin H (Cat H) are involved in proteolytic processing and activation of granzymes, main effector molecules of CD8+ cytotoxic lymphocytes whereas Cat L has been implicated in processing and activation of perforin.

Osteoblasts represent the major cell type of the HSC niche. Although the role of osteoblasts in supporting HSCs was shown to be more limited than previously expected [reviewed in more detail in (91)], they play an important role in immune regulation through secreting biologically active molecules that maintain the BM microenvironment and factors such as CXCL-12 necessary for stem cell maintenance (90) (**Figure 1**). Osteoblasts represent a rich reservoir of cathepsins and were shown to secrete proteolytically active cysteine cathepsins, such as cathepsins B, K, L, and X (68). All of these cathepsins were shown to digest the chemokine CXCL12, a key mediator that retains hematopoietic progenitor cells in their niches (69). Furthermore, only CatX can inhibit adhesive interactions between CD34^+^ hematopoietic progenitor cells and osteoblasts (70) (**Figure 2**). Interestingly, whereas CatX is preferentially expressed by mature cells of the hematopoietic and immune system, it is not synthesized by hematopoietic progenitor cells (70).

Osteoclasts are derived from the same myeloid precursor cells that give rise to macrophages and myeloid DCs. Their differentiation is marked by increased CatK expression and downregulated expression of cathepsins B, L, and X (92). Compared to wild-type (*wt*) mice, CatK(−/−) mice exhibited significantly increased numbers of committed CD11b(+) osteoclast precursors in the BM, whereas the percentage, but not the number of these cells, was decreased in the spleen (93). Additionally, osteoclast precursors lacking CatK fail to migrate and engraft in a fracture wound during injury repair, indicating that CatK is also required for osteoclast engagement in response to microenvironmental stimuli (66). Increased CatK expression in mouse leads to increased osteoclast formation from BM precursors *in vitro* (71). Initial studies on mice with CatK deficiency demonstrated abnormalities in hematopoietic compartments, decreased BM cellularity, and splenomegaly (66). A later study showed that a CatK deletion in mice resulted in reduced numbers of immature lineage-SCA1+ KIT+ (LSK) cells and mature Ly6Clow and CD11c+ cells in BM (93). However, until recently, the physiological role of CatK in stromal cells of the HSC niche, other than osteoclasts, has not been explored. Stromal cells that maintain HSCs during *in vitro* culture show high transcription of the CatK gene. Although no effects of CatK loss on early hematopoiesis or myelopoiesis were detected *in vivo*, *in vitro* cultivation of LSK cells on the CatK-deficient HSC supportive stromal cell line UG26-1B6 (94, 95) lead to reduced colony formation and proliferation and increased differentiation to myeloid progenitors (96). The subsequent transfer of LSK cells generated in a CatK-deficient microenvironment into *wt* mice revealed reduced donor LSK repopulating activity and lymphopoiesis maintenance (96). Further transplantation experiments revealed that environmental CatK is particularly important for the optimal regeneration of B- and T-lymphocyte compartments in different hematopoietic tissues. The most pronounced changes were detected in T lymphopoiesis in BM, implying that early T-cell development critically depends on CatK (96).

In addition to CatK, its closely related member of the papain family, CatL, has also been shown to regulate lymphopoiesis in BM. CatL deficiency was shown to affect B-cell production by acting both on BM stem cells and BM B-cell precursors (72). Compared to *wt* mice, mice with *CTSL* gene mutations (73) exhibited unaltered percentages and absolute numbers of pre-pro-B, pro-B, pre-B, and immature and mature B cells in BM but increased B-cell production and emigration from BM, leading to increased peripheral B-cell numbers (72). These data implicate CatL as a negative regulator of BM B-cell production and output, therefore influencing the homeostasis of peripheral B cells. Furthermore, in BM, pro-B cells are continuously produced and die unless they express pre-B cell receptor (pre-BCR) (74). One of the proposed mechanisms of removing pro-B cells carrying non-productive variable (V), joining (J), and diversity (D) gene [V(D)J] rearrangement is through Toll-like receptor (TLR)-9 expressed in cells of the innate immune system as well as in B lymphocytes and their progenitors (97). TLR9 signaling directly inhibits the expansion of pro-B cells *via* a CatB-dependent mechanism that promotes caspase-independent cell death (97). This regulatory mechanism is proposed to regulate lymphopoiesis *in vivo* and to participate in the homeostatic control of precursor compartments.

Cathepsins are most known for their involvement in degrading antigens (endocytosed as well as endogenous antigens) to antigen peptides that bind to the MHC II. A precondition for this to happen is the degradation of the invariant chain (Ii) chaperone that is associated with MHC class II molecules; this is also achieved by cathepsins (98). The Ii chaperone associates with MHC class II molecules immediately after their synthesis in the endoplasmic reticulum; the complex is then transported to endosomes and lysosomes in which it comes into contact with degraded antigens (99). Once bound to MHC class II molecules, antigen peptides are transferred to the membrane of APCs (100). The involvement of CatL in thymic CD4+ T-cell positive selection has been well documented (**Figure 1**). CatL is critical for MHC class II-mediated peptide presentation in thymic epithelial cells, acting both in Ii degradation (101) and in the generation of MHC class II-bound peptide ligands presented by cortical thymic epithelial cells (102). Consequently, CatL-deficient mice exhibit impaired CD4+ T-cell selection, an altered repertoire of CD4+ lymphocytes, and an increased number of CD8+ lymphocytes (102). Cat V, that shares 80% protein sequence identity with Cat L is exclusively expressed in the thymic cortex in humans and it is proposed to have overtaken the role of Cat L in the positive selection of thymocytes (**Figure 2**). This is further supported by its ability to selectively degrade Ii complexed to MHC (103). Mice with the mutated *CTSL* gene (73) exhibit hypertrophied lymph nodes with normal absolute numbers of CD4+ and increased numbers of CD8+ lymphocytes (104). In the same way as Cat K, a proportion of CatL is physiologically secreted and can be extracellularly activated (76) after which it can process several ECM proteins, such as fibronectin, laminin, elastin, and collagen types I, IV, and XVII (76, 77, 105–107). Furthermore, ECM components have been shown to transduce survival signals (108) and promote the proliferation of lymphocytes by signalling through integrins (109). By regulating the level of expression of ECM components in lymphoid organs, CatL can have a broad effect on immune system (104). In lymph nodes of CatL-deficient mice ECM components such as laminin, fibronectin, collagen I and IV were found to be increased. Furthermore, enlarged lymph nodes (LN) from mice with mutated *CTSL* gene (73) contained increased numbers of mature B cells due to their higher input into the LN (72). Apart from being a key regulator of classical MHC II antigen presentation in the thymus, CatL is also essential for nonclassical CD1d presentation (110). The absence of CatL expression in thymocytes abrogates their capacity to stimulate autoreactive NKT cells *in vitro* and precludes their selection *in vivo* (110). Furthermore, the maturational transition of NKT cells requires continuous T cell receptor (TCR)/CD1d interactions with the components of the thymic cortex. A recent study demonstrated that key components necessary for positive NKT cell selection, such as CatL, are also required for subsequent NKT cell maturation (111).

### Immune Cell Subset Differentiation

Apart from their role in T lymphopoiesis, cathepsins have been implicated in T-cell lineage commitment. Unlike thymocytes, two major T helper (Th1 and Th2) lineage cells do not express CatL (112, 113). However, in addition to being regulated by cytokines and transcription factors, the differentiation of CD4+ cells to Th17 cells is actively regulated by CatL in mice (113). Mouse Th17 cell differentiation *in vitro* was suppressed by both broad cathepsin inhibitors and CatL selective inhibitors (113). Tuomela et al. identified *CSTL* among genes upregulated at the early stage of Th17 cell differentiation (114). Recently, CatL was characterized as a promising marker for human Th17 cell identification (112). Furthermore, CatK regulates signaling pathways proximal to TLR9 (115) and TLR7 (116) in DCs and affects Th17 polarization. Besides CatL and CatK, CatS plays a critical role, after exposure to components of periodontal bacteria, in driving splenic DC-dependent Th17 differentiation by upregulating IL-6 through activating DC protease-activated receptor-2 (PAR2) (117) (**Figure 2**). Finally, CatC was also implicated in cathepsin-dependent lysosome disruption in myeloid leukocytes and Th2-associated immune response, after adjuvant treatment (118). Cathepsins have also been implicated in the regulation of the activities of T regulatory cells (Tregs). Apart from being involved in TLR9 and TLR7 signaling in DCs, it was recently demonstrated that CatK produces cleaved TLR7 *in vitro*, and in Tregs implicating CatK in blocking Treg immuno-suppressive activity and in promoting the development of murine systemic lupus erythematosus-like manifestations (119) (**Figure 2**).

In APCs, the alterations in cathepsin expression, activity, or localization are closely related to their maturational and functional state. The differentiation of macrophages and DCs from granulocyte/macrophage progenitors is marked by a significant surge in cathepsin expression (7, 80). Furthermore, cytokine-stimulated HSCs, which were primed either towards immunostimulatory or immunotolerant DC phenotypes, showed differential patterns of CatS expression and activity (120). Recent data indicate that cathepsins also induce the switch between two distinct functional states of macrophages. CatL and CatS were shown to support M2 (anti-inflammatory) (121, 122), whereas CatC was shown to support M1 (pro-inflammatory) macrophage polarization through FAK-triggered p38 MAPK/NF-κB pathway (123, 124).

### Immune Cell Homeostasis

At the end of an immune response, the contraction of the T-cell response results in massive apoptosis with a small subset of highly specific memory cells surviving. Once the memory T-cell population has been established, its long-term maintenance depends on successful survival and homeostatic proliferation in BM. As different pathogens are encountered, memory CD8+ T-cell populations from earlier infections (which have not re-encountered antigen) decline in frequency (125). A recent study in mice showed that lysosome stability and pro-apoptotic CatB activity limits the long-term maintenance of memory CD8+ T-cell populations. The serine protease inhibitor (Spi) 2A, an anti-apoptotic cytosolic cathepsin inhibitor that is induced by IL-15 and IL-7, was found to be necessary for counteracting pro-apoptotic CatB activity for the maintenance of memory CD8+ T lymphocytes (126) (**Figure 1**).

Autophagy is highly regulated lysosome-dependent process necessary for homeostasis quiescent cells of the immune system (82). Namely, memory B and T cells, plasma cells and tissue resident macrophages, as well as HSCs, all require autophagy for their homeostasis (82). Cat B genetic ablation in murine Bone Marrow-Derived Macrophages (BMDM) resulted in a nearly 2-fold increase in the number of lysosomes as well as in enlargement of both single membrane lysosomes and double-membrane autophagosomes compared to wild type cells (127).These results suggested that Cat B negatively regulates lysosomal biogenesis and autophagy (**Figure 2**). This is at least partially achieved through degradation of lysosomal calcium channel mammalian mucolipin TRP channel 1 (TRPML1) and alteration in lysosomal calcium signaling and regulation of transcription factor EB (TFEB) activity by Cat B (127).

Furthermore, through cleavage of talin, that critically controls integrin-dependent cell migration, CatH (31) could be involved in maintaining the homeostasis and survival of the regulatory T (Treg) cell pool (128).

β2 integrins in innate and adaptive immune cells are group of receptors crucially involved in leukocyte differentiation, activation/polarization, and functional activity (84, 129).

Pro- and mature forms of cathepsin X interact with β2 integrin receptors, thus modulating their affinity for extracellular ligands (130) (**Figure 2**).

## Endogenous Cathepsin Inhibitors

Cathepsin activity must be controlled at several levels to prevent severe tissue damage. Cathepsin expression is spatially and temporally regulated and, as mentioned above, cathepsins are expressed in the inactive form as proenzymes. After activation, endogenous protein inhibitors are important for controlling proteolysis [summarized from (131)].

Endogenous inhibitors mainly bind, reversibly or irreversibly, into the active site of the enzyme. Based on the properties of their action, they are divided between emergency and regulatory inhibitors (132). Emergency inhibitors rapidly bind peptidases into a stable complex and prevent their further action. Their concentration exceeds the amount of their target peptidase, and they are usually spatially separated from their target peptidases. They act on the peptidases that left their natural location and on peptidases released from invading microorganisms. Accordingly, regulatory inhibitors are localized with their target enzymes. Their task is not to prevent but to regulate peptidase activity. They are further divided into three types. 1) Threshold inhibitors act similarly to emergency inhibitors but with a much lower concentration. 2) Buffer type inhibitors bind quickly to the enzyme when no substrate is present and are also quickly released in the presence of the substrate. They prevent harmful proteolysis. N-terminal regions of cysteine peptidases, for example, can also act as buffer inhibitors. After removal during peptidase activation, they operate as potent reversible inhibitors of their parent enzymes and can be referred to as propeptide-like inhibitors. The proregion, by covalently binding to the N-terminal of the mature enzyme, physically obstructs access to the active site groove. Proregions exhibit high selectivity toward their parent enzymes [summarized in (133)]. 3) Delay type inhibitors act irreversibly or pseudo-irreversibly by slowly binding to the peptidase after its activation. In that way, they allow peptidases to act for a certain period. The role of endogenous inhibitors is thus not only inhibiting peptidases but also regulating their stability and expression and preventing infection by inhibiting exogenous peptidases from microorganisms. Finally, endogenous inhibitors also act through ways other than peptidase inhibition (132). They belong either to the cystatin or thyropin family.

### Cystatins

In the past few decades, the cystatin group of peptidase inhibitors, classified as peptidase inhibitor family I25 (134), has been reviewed many times [(135–137), to list just a few]. Here, we will thus only summarize the typical characteristics of each type, while their role in immune cell functions will be discussed in the chapter below. Cystatins are protein inhibitors that relatively non-selectively inhibit cysteine peptidases. However, they are more potent inhibitors of cysteine endopeptidase activity than exopeptidase activity [reviewed in (132)]. For example, CatX, a carboxymonopeptidase, is not inhibited by any cystatin (138). Two conserved regions are necessary for cathepsin inhibition: the central QXVXG region and the P-W pair in the C-terminal part of cystatins (135, 136). Cystatins are competitive inhibitors, which bind tightly and reversibly to their targets and inhibit their targets in the μM to pM range (135). In the MEROPS database, cystatins are divided into four groups based on the copies of cystatin-like sections and the presence or absence of disulfide bonds (139).

### *Type I Cystatins* (The Stefin Family)

Stefins are single-chain proteins with molecular masses of around 11 kDa and possess no disulfide bonds or carbohydrates. They are located intracellularly in the cytoplasm or extracellularly in body fluids. They are stable at neutral and alkaline pH and are also heat-stable (135). In humans, stefins A (also cystatin A) and B (also cystatin B) are well known. They potently inhibit papain and cathepsins L, S, and H. Additional stefins have been characterized in mice, cattle, and pig [summarized in (136)].

### *Type II Cystatins* (The Cystatin Family)

These cystatins are single-chain proteins with molecular masses of 13–15 kDa. They possess two disulfide bonds and are usually non-glycosylated, except for cystatins F and E/M, which are glycoproteins. They are present at higher concentrations mainly extracellularly in biological fluids such as seminal plasma and cerebrospinal fluid, whereas their concentrations are lower in plasma, saliva, and urine. Type II cystatin inhibition of cysteine peptidases is the strongest among the cystatins; Ki values for endopeptidase activity inhibition can reach as low as the fM range [reviewed in (136)]. In humans, the cystatins C, E/M, F, D, S, SA, N, and SN are expressed in different tissues. However, the first described cystatin was isolated from the chicken egg-white and was named chicken cystatin (140).

### *Type III Cystatins* (The Kininogen Family)

Kininogens are single-chain multifunctional glycoproteins with molecular masses of around 120 kDa for high molecular weight kininogen or around 68 kDa for low molecular weight kininogen. They are stable at neutral and alkaline pH and are also heat stable [summarized in (135)]. They are present in mammalian plasma and secretions. They have three cystatin-like domains of which only two have inhibitory properties. They strongly inhibit papain and CatL and weakly inhibit cathepsins H and B (136). Besides their inhibitory activity, they are large precursors of the vasodilator kinin peptides bradykinin and kalidin, which participate in blood pressure regulation (141) and can act as antimicrobial peptides (through their fifth domain), inducing apoptosis of proliferating epithelial cells (142).

### Thyropins

Thyropins (thyroglobulin type-1 domain proteinase inhibitors) are proteins with one, two, or three thyroglobulin type-1 domains that express inhibitory action toward cathepsins, mostly cysteine cathepsins (143). They are classified as peptidase inhibitor family I31 (134). The thyroglobulin type-1 domain was first identified and characterized in thyroglobulin. It possesses four or six cysteine residues and two patterns: the Q-C pattern at consensus positions 26-27 and the C-W-C-V pattern at positions 38-41. A databank search located similar domains in 29 proteins with different functions, unrelated to thyroglobulin (144). They can be either peptidase inhibitors, substrates (145), or both (146). Thyropins bind tightly and reversibly to cysteine peptidases (147). Several thyropins have been characterized in humans: the p41 fragment of the Ii (147), testicans 1 (148) and 3 (149), and nidogens/entactins 1 and 2 (138, 150). Besides human thyropins, also equistatin from the beadlet anemone *Actinia equina* (151), saxiphilin from the bullfrog *Rana catesbiana* (152), and the chum salmon (*Oncorhynchus keta*) egg peptidase inhibitor (150) were described as inhibitors of papain and cathepsins B and L, while equistatin can also inhibit aspartic cathepsin D (153).

The p41 fragment of the invariant chain (Ii) is expressed in two splicing forms, p31 and p41, which differ in the presence of the additional fragment of 64 amino acids in p41 that includes the thyroglobulin type-1 domain (147). The p41 fragment inhibits cathepsins L and H as well as papain and cruzipain (147, 154). It also plays an important role in antigen presentation, as will be discussed later.

Testicans are extracellular chondroitin/heparan sulfate proteoglycans that were first identified in seminal plasma (155). Three homologues have been found in humans, which share 40–50% homology and are mostly expressed in the central nervous system, named testicans 1, 2, and 3 (149). Their multidomain structure includes several domains that indicate that they may act as inhibitors. They possess three domains: 1) a domain that inhibits membrane-type 1 matrix metalloproteinase (MT-MMP) activation of MMP-2, 2) a Kazal-like domain present in serine peptidase inhibitors, and 3) the thyroglobulin type-1 domain [summarized in (148)]. Testican 1 is a competitive inhibitor of CatL at acidic (5.5) and neutral (7.2) pH. Protein-protein interactions are needed through which testican 1 not only inhibits CatL but also enhances its stability at neutral pH; the enzyme’s activity is thus prolonged at the price of reduced proteolytic activity. Inhibiting MTbuffer1-MMP and MT4-MMP, which are important for the processing of MMP2, testicans 1 and 3, and the splicing variant of testican 3, N-Tes (which lacks the thyroglobulin domain), prevents MMP2 activation (149). Testican 2 has not been shown to inhibit cathepsins.

The nidogens/entactins 1 and 2 are ubiquitous components of basement membranes and essential for morphogenesis before and around birth (156, 157). Mouse entactin does not show any inhibitory activity toward papain (150). Human thyroglobulin domain 1 of nidogen 1 showed inhibitory activity toward cathepsins L and B with 100-fold molar excess over the enzyme ratio (138).

### Serpins

Serpins, typical inhibitors of serine peptidases, can also inhibit cysteine peptidases through cross-class-specific inhibition  (158). Cross-class-specific intracellular serpins were found to inhibit papain-like, lysosomal cysteine cathepsins, such as cathepsins B, V, L, K, and H (158). For example, serine peptidase inhibitor 2A (Spi2A or Serpina3g) inhibits the serine peptidase cathepsin G but also papain-like, lysosomal cysteine cathepsins such as cathepsins B, V, L, K, and H (159). This cross-class specificity of Spi2A for cysteine cathepsins is also a property of another mouse serpin, SQN-5 (Serpinb3a) (160). Similarly, human squamous cell carcinoma antigen-1 is a potent inhibitor of the cathepsins K, L, and S (161). The serpin hurpin specifically inhibits only CatL in human keratinocytes  (162). The mechanism of inhibition of cysteine peptidases involves cleavage of the intracellular serpin through a stable covalent (158, 160) or non-covalent (159, 163) complex. Intracellular serpins are involved in an inhibitory pathway that regulates a powerful array of lysosomal cysteine proteinases that trigger cell death. As such, there is an emerging view that these serpins are important regulators of cell survival.

## Regulation of Immune Cell Functions by Cysteine Cathepsins And Their Inhibitors

Immune cell functions are tightly regulated by cathepsins and their endogenous inhibitors. The ratio of both is of extreme importance for normal physiological performance of the immune system (99, 164). The most studied processes are antigen processing and the presentation of processed fragments in APCs, the cytotoxicity of CTLs and NK cells, and the protection of the host from invading pathogens (165). As cathepsins perform diverse actions in different cells, we will review their involvement by focusing on cysteine cathepsins.

### Antigen Presentation

DCs are professional APCs that, in their immature form, detect, engulf, and process pathogens. When activated, they lose their ability of capturing and processing and instead migrate to the secondary lymphoid organs in which they present antigens on their surface to prime B and T lymphocytes. As such, DCs connect the innate and adaptive immune responses (166, 167).

In DCs, CatS is the major cysteine cathepsin involved in processing and presenting antigens (168) and its activity increases during DC maturation (**Figure 2**). This is not due to increased CatS expression but to the changes in the expression and localization of its inhibitor, cystatin C. At the same time, step-wise Ii processing also increases (164); this prevents the targeting of MHC class II molecules to lysosomes in which they would degrade. CatS plays a similar role in other BM-derived APCs, while in cortical thymic epithelial cells, CatL was found to be the preferred cysteine cathepsin that is also regulated by cystatin C (101). Other cathepsins, cysteine as well as others (B, D, E, and legumain, also known as asparaginyl endopeptidase) also participate but are not essential in antigen processing (13).

The expression of cystatin C in immune cells depends on their differentiation status and maturity (169). High cystatin C levels are found in lysosomes of immature DCs; however, cystatin C levels significantly decrease during DC maturation (170, 171). Cystatin C expression varies between different DC subsets, and thus it may not be necessary nor sufficient to control MHC class II molecule expression and antigen presentation. Furthermore, in cystatin C knockout mice, mature DCs displayed no defect in MHC class II molecule surface expression (172). However, cystatin C can influence the expression of surface MHC class II molecules through decreased expression of chaperone H2-DM (173) and thus inhibits CD4+ T-cell proliferation. It can also stimulate DCs to secrete more of the immune suppressive cytokines IL-10 and transforming growth factor β (TGFβ) and less of the pro-inflammatory cytokine TNFα (174). It also inhibits antigen presentation by MHC class I molecules that leads to compromised activation of CD8+ T cells (174). To sum up, the mechanisms underlying cystatin C action, including its role in antigen presentation, are far from understood. Infection with *Ectromelia virus* in murine conventional DCs, leads to downregulation of cathepsins B, L, and S and cystatins B and C, diminishing their ability to process antigen, that results in induction of insufficient adaptive immune response (175). Additionally, cystatin D (176) and cystatin F (177) seem to play a role in antigen presentation.

### Cytotoxicity of CTLs and NK Cells

CTLs and NK cells are major effector cells of the immune system, killing their targets through the perforin/granzyme pathway. After recognizing a cell that has been infected by a virus or transformed, they release the content of their cytotoxic granules into the lytic synapse between the killer cell and target cell. Cytotoxic granules consist of a plethora of proteins, including perforin, serine peptidase granzymes, and cathepsins C, H, and L (178). Perforin is a pore-forming protein that enables the entry of granzymes A and B into the target cell. Granzymes A and B then trigger apoptosis through different signaling pathways, both with or without activation of caspases (179, 180). Perforin is activated proteolytically by CatL. In CatL-deficient NK cells from mice, a small amount of perforin is still processed (181). CatC activates both granzymes by proteolytic cleavage (182), while in the absence of CatC, CatH takes over by activating granzyme B. Still, when lacking both cathepsins, mice still retain low activity of granzyme B, indicating the involvement of yet another peptidase (183) (**Figure 2**). Legumain may also play a role in NK cells, as they display lower cytotoxic activity in legumain-null mice (184). Cysteine cathepsins from cytotoxic granules as well as the tight regulation of their activities are thus indispensable for the killing functions of CTLs and NK cells. Cystatin F is the most important candidate for such regulation.

Cystatin F is expressed as an inactive dimer and first needs to be converted to its monomeric form (185). As a monomer, it is a potent inhibitor of cathepsins F, K, L, and V and to a lesser extent of cathepsins H and S (185). However, if the monomers are further processed on the N-termini, truncated cystatin F becomes a strong inhibitor of CatC (186). Another distinct feature of cystatin F is that it can also function *in trans*. After secretion, it is taken up by surrounding cells (187) in which it is then activated within endo-lysosomal vesicles. Here, cystatin F inactivates cathepsins C and H, which leads to reduced cytotoxicity of these cells (180, 188). Furthermore, cathepsins B and S (189) have been implicated in the activation of invariant NKTs, capable of recognizing lipids in an antigen-specific manner and rapidly secreting high amounts of cytokines upon antigenic stimulation.

### Protection of the Host From Invading Pathogens

Eosinophils are effector cells of the immune system that are imperative for the defense against nematodes and other parasites. They also participate in the inflammatory response of allergic reactions (190). Eosinophils have abundant intracellular granules containing major basic proteins, other highly toxic proteins, and cathepsins S, B, X, C, and L, which process major basic proteins and other proteins during granule biogenesis. Cystatin F was reported to be a survival factor for eosinophils, especially for eosinophils with high granule content. It regulates the activities of cysteine peptidases, which are critical for normal granule biogenesis. Furthermore, cystatin F also protects eosinophils from being harmed by their toxic proteins and is thus essential for eosinophil viability (191). Cystatin SN is another endogenous inhibitor that is important for eosinophil immunity. It is involved in the activation and recruitment of eosinophils during eosinophilic inflammation. Cystatin SN derived from epithelial cells induces IL-5 production that promotes the synthesis of granule proteins, and cystatin SN expression is controlled by several cytokines (192).

Neutrophils are effectors of the rapid innate host defense response against bacteria and fungi. They can promptly migrate to sites of inflammation, engulf foreign bodies, and produce several microbicidal substances such as superoxide [summarized from (193)]. CatC activates certain serine peptidases that are found in mature neutrophils such as cathepsin G, proteinase 3, and neutrophil elastase (194). CatS activates CatC in a stepwise manner in neutrophilic precursor cells. Neutrophils are also the source of CatC (195). Conversely, cystatin C plays a role in neutrophil migration through modulating chemotaxis and regulating the phagocytic function of polymorphonuclear neutrophils (196).

Monocytes and macrophages actively participate in the innate immune response. They recruit and activate other immune cells by secreting a wide range of pro-inflammatory (M2-polarized macrophages; IL-12, TNFα) and immunomodulatory (M1-polarized macrophages; IL-4, IL-10) cytokines and chemokines [summarized from (197)]. During differentiation from monocytes to macrophages, the intracellular proteolytic profile significantly changes. Even though the expression levels of cathepsins L, S, and C, and legumain decrease, their activities increase in macrophages because the precursor-to-active form ratio stays the same. This is due to the absence of cystatin F that is mainly present in monocytes but not in macrophages. One exception is CatH with unchanged activity (198). The expression of cystatin F is regulated by transcription factor C/EBPα, whose expression declines during monocyte differentiation (78). C/EBPα binds to the promoter region of the cystatin F gene, and this binding decreases after differentiation (198).

Cystatin C is expressed constitutively in monocytes/macrophages (79) and influences the phenotype of monocytes (199). During bone formation, it reduces osteoclastic activity by inhibiting cathepsins and is as such required for macrophage-mediated regulation of bone cells (199). Next, the expression of cystatin C increases during the differentiation of monocytes to immature DCs (170). In microglia (resident macrophages of the nervous system), cystatin C modifies migration, possibly by neutralizing ECM remodeling cathepsins (81). During the differentiation of monocytes to macrophages, stefin B mRNA increases (200). Stefin B was proposed to play a role in the innate immune response to bacterial infection (201).

In general, one or more cathepsins and their inhibitors are involved in every step of the immune response, from pathogen entrance to the final immune effect. Cathepsins activate TLRs. Cathepsins B, L, S, and F were shown to cleave TLR-7 and -9 (202). They also activate or inhibit different cytokines and are themselves regulated by cytokines (203, 204). CatB is needed for the processing and production of TNFα in response to bacterial lipopolysaccharide (LPS). It obstructs the trafficking of TNFα-containing vesicles to the plasma membrane (205). The expression of cystatin C increases after LPS-induced stimulation of monocytes (201). CatB reduces the production of the proinflammatory cytokines IL-1b and TNFα by decreasing ERK1/2 phosphorylation that leads to down-regulated transcription of certain cytokines (199). The same study also showed that cystatin C induces the release of the proinflammatory cytokines IL-6 and IL-8 in non-pathogenically activated monocytes. Cystatin C in DCs suppresses DC-induced differentiation of T cells that have more anti-inflammatory potential (174). Cystatin C is possibly also involved in the regulation of complement activation because it binds to complement factor C4 (206).

CatX regulates the migration, proliferation, maturation, phagocytosis, signal transduction, and adhesion of DCs, monocytes, and macrophages. As a carboxymonopeptidase, it acts through proteolytic cleavage of the C-terminus of integrin receptors or through its tripeptide motif Arg-Gly-Asp (RGD) (44, 207). Thus, it can indirectly also affect the function of cytotoxic cells. Although cystatin F cannot inhibit CatX, it affects CatX (138, 185) by inhibiting CatL, which is necessary for the activation of CatX. In DCs, cystatin F possibly regulates the activity of CatL, thus controlling the processing of pro-CatX, which promotes cell adhesion (208).

## The Role of Cathepsins and Their Inhibitors in Inducing Tolerance and Anergy in Immune Cells

Cathepsins are also involved in the induction of apoptosis in immune cells as a mechanism to regulate inappropriate immune responses. In a model of Ag-specific unresponsiveness, i.e., high dose tolerance, the incubation of T cells with increasing anti-thymocyte globulin concentrations *in vitro* reduced lymphocyte proliferative responses. This was associated with a rapid increase in the percentage of apoptotic cells as a consequence of cytosolic CatB activity (209). Similarly, the exposure of peripheral blood monocytes to increasing mitogen concentrations reduced the proliferative response and release of CatB and CatL into the cytosol as a consequence of LMP (210). CatV was implicated in promotion of the maturation of thymocytes, including those with an autoimmune potential and increased expression of CatV has been found in thymi of *Myasthenia Gravis* patients (103). Finally, the increased expression of CatS involved in MHC II-dependent antigen presentation in DCs was shown to lead to improper antigen presentation, associated with the development of autoimmunity (211) (**Figure 1**).

In germinal centers, B lymphocytes with high-affinity B-cell receptors are selected and B-cells with unwanted specificities are eliminated. Apoptotic death in germinal center B lymphocytes can be blocked not only by inhibition of caspase-8 or caspase-3 but also by inhibition of cathepsins (212). This antigen-dependent germinal center B cell death was shown to be a consequence of LMP and release of cathepsins B and L into the cytosol, whereas the interaction with follicular DCs and CD40 ligation was shown to rescue B cells from LMP and apoptosis (213).

NK cells lose the ability to mediate cytotoxicity and down-regulate CD16 receptor expression upon interaction with cancer stem cells (CSCs)/undifferentiated tumors or through cross-linking of CD16 receptors by antibodies, while maintaining secretion of IFN-γ and TNF-α. This functional state of NK cells, termed “split anergy” is characterized by a selective loss or decrease in cytotoxicity and an increase in cytokine and chemokine production (214). An *in vitro* study showed that this loss of NK cell cytotoxicity is a consequence of decreased activity of the key granzyme-activating cathepsins C and H and increased concentration of their endogenous inhibitor, cystatin F (215). Similarly, the induction of functional anergy in CD8+ T lymphocytes using ionomycin or TGFβ decreased the activity of cathepsins C and H and increased the concentration of cystatin F (180). Cystatin F is only partially targeted to endo-lysosomes upon its synthesis, and can also be secreted and taken up by surrounding cells (188). The uptake of exogenous cystatin F was also shown to reduce the cytotoxicity of NK cells and T cells (188, 216). Furthermore, proinflammatory cytokines, such as IFN-γ and TNF-α, increase cathepsin S and B activities in DCs (217, 218), whereas IL-10, which induces tolerogenic DCs and consequently T lymphocyte anergy (219), prevents the upregulation of cathepsin S and B activities in DCs (220). The changes in CatS activity in activated DCs could be, at least in part, attributed to changes in endo-lysosomal levels of its endogenous inhibitor, cystatin C (164).

## The Role of Cathepsins and Their Inhibitors in Immunosenescence

Age-related declines in the immune system, known as immunosenescence, result in increased susceptibility to infectious diseases, tumors, and autoimmune diseases as well as impaired responses to vaccination in older individuals. The deterioration of the immune response during aging can at least partially be attributed to changes in the major cellular proteolytic mechanisms mediated by the lysosome (221). During senescence, lysosomes undergo various changes that decrease their degradative capacity. Age-associated increases in the size and numbers of lysosomes (222) as well as in the enhanced fragility and increased LMP in response to various stimuli (223) have been well documented.

CatK serum levels were shown to decrease with age (224). Furthermore, aging affects the composition of the ECM, and the aging-associated changes of collagen fibers were suggested to influence the collagenolytic activity of CatK. The accumulation of advanced glycation end-products and mineralization were all shown to reduce CatK collagenolytic activity, while the removal of GAGs completely blocked this activity (225). The amount of chondroitin sulfate, a major subtype of GAGs in bone, also decreases with ageing (226).

Whereas tightly regulated, short-term, acute inflammation is a normal defense mechanism that acts against harmful agents, dysregulation of the immune response leads to chronic systemic inflammatory states. Age-associated chronic inflammation is characterized by unresolved and uncontrolled inflammation that exacerbates the aging process and age-related chronic diseases. Among the dysregulated proinflammatory cytokines and chemokines, elevated expression and secretion of cathepsins is an important culprit underlying the development and progression of cancer, autoimmunity, atherosclerosis, neurodegeneration, and osteoporosis (14, 32, 227) with age. For example, a rich source of cathepsins in the brain are activated microglia (228). They can comprise up to 20% of the total glial cell population. Microglia-secreted cathepsins, such as CatS and CatX, were found to be upregulated in mouse microglia with aging (229, 230). Activated microglia were suggested to be involved in neuroinflammation that accompanies numerous neurodegenerative disorders, including Alzheimer’s disease.

The age-associated decline of T-cell function is complex and occurs at multiple levels. Whereas maintaining memory cells is of utmost importance for subsequent encounters with antigens, senescent CD8+ T cells accumulate over time and can constitute > 50% of the peripheral blood CD8+ T-cell pool in the elderly due to their resistance to apoptosis (231). The accumulation of senescent CD8+ T cells influences the quality of the memory T-cell pool, impairs the capacity to respond to vaccination, and is highly associated with age-related diseases (231). A recent study showed that *Serpina3g* (that encodes Spi2A and is necessary for counteracting the pro-apoptotic effects of CatB and the maintenance of long-term memory CD8+ T cells) expression was increased in both old CD8+ central memory (TCM) and effector memory (TEM) cells, supporting the long-term survival of CD8 T memory cells (232). Furthermore, a cohort of healthy elderly volunteers exhibited no differences in CatB activity in their granulocytes, monocytes, or lymphocytes but exhibited increased CatC (DPP I) activity in their lymphocytes, compared with young controls (233). This is in line with data suggesting that a high proportion of cytotoxic cells, as well as proper cytotoxic functions during senescence, are determining factors for healthy ageing (234).

## Cathepsins and Their Inhibitors as Chemotherapeutic Targets for Modulating Immune Cell Functions

Pharmacological inhibition or targeted disruption of cathepsins has been widely studied in the context of cancer and pathological conditions involving bone and cartilage. With few exceptions, the potential of cathepsins in regulating and regenerating immune cell function is only beginning to be explored (**Table 2**).

**Table 2:** The effects of cysteine cathepsin activity modulation on immune cell subset differentiation.

*T lymphocytes*	
Th1	Treatment of *Leishmania major* -infected BALB/c mice with a specific inhibitor of Cathepsin B (CA074) augmented the Th1 response (232).
Th2	Treatment of *Leishmania major* -infected BALB/c mice with a specific inhibitor of Cathepsin L (CLIK148) , enhances the development of Th2 response (235).
Th17	Theatment with specific Cathepsin L inhibitor inhibits differentiation of Th17 T cells (106).
	Cathepsin K inhibition decreased Th17 cell induction in response to unmethylated CpG DNA thorough defective TLR 9 signaling in DCs (108).
	Cathepsin S-induced IL-6 production by splenic DCs promotes Th17 differentiation, in response to systemic exposure to LPS derived from *Porphyromonas gingivalis* (PgLPS) in mice (110).
Tregs	Cathepsin S inhibition under influence of tumor cells inhibits Treg immunosupressive activity (226).
***Macrophages***	
M1	Increased Cathepsin C activity promotes macrophage M1 polarization trough FAK-induced p38MAPK/NF-κB signaling pathway (117).
M1	Inhibition of cathepsins B, L, and S with GB111-NH2, leads to polarization shift from M2- to M1 macrophages (231).
M2	Cathepsin S knockout inhibited M2 macrophage polarization during tumor development (116).

TLR9, Toll-like receptor 9; FAK, Focal Adhesion Kinase; MAPK, mitogen-activated protein kinase; NF-kB, Nuclear factor-kB; DCs, dendritic cells; Tregs, regulatory T cells.

Due to its central role in ECM degradation and bone remodeling, CatK is currently considered one of the most promising targets for the treatment of osteoporosis (235). Increased CatK levels were found in synovial fluids and the lining tissue inside arthritic joints from patients with rheumatoid arthritis, implicating CatK in the development and/or progression of this disease (236). Pharmacological inhibition or targeted disruption of CatK resulted in defective TLR9 signaling in DCs in response to unmethylated CpG DNA, which in turn led to an attenuated induction of Th17 cells. These data suggest that CatK may serve as a valid therapeutic target in autoimmune diseases (115). Specific inhibitor of CatS (Clik60) *in vitro* markedly impaired presentation of an organ-specific autoantigen, 120-kDa alpha-fodrin, by interfering with MHC class II-peptide. Treatment with CatS inhibitor *in vivo* was effective in preventing the development of autoimmune lesions in the salivary and lacrimal glands of the Sjögren syndrome model mice (237).

*In vitro* studies using targeted disruption of cathepsins identified cathepsins B, C, L, and S as potential modulators of immune cell activity in diseases such as cancer. Whereas CatS inhibition enhances immunosuppressive activity of Tregs under normal conditions, transfer of CatS inhibitor-treated Tregs into bladder carcinoma MB49 model mice led to reduction in number of splenic and tumor Tregs, and reduction of tumor and splenic cell proliferation compared to control animals (238). The tumor microenvironment is particularly conducive to the development of M2 macrophages and myeloid-derived suppressor cells, which potently suppress the anti-cancer immune response. In comparison to their anti-tumor counterparts, these cells significantly upregulate the expression and activity of cysteine cathepsins (75, 239). Blocking these cysteine cathepsins could inhibit their immunosuppressive functions, subsequently alleviating the tumor burden. Experiments *in vivo* support this rationale. Genetic ablation of CatB successfully prevented the accumulation of myeloid-derived suppressor cells and attenuated polyposis in a mouse APC^∆468^ model (240). GB111-NH_2_, a broad-spectrum inhibitor of cathepsins B, L, and S, which was applied to 4T1 tumor-bearing mice, induced tumor regression as a result of macrophage apoptosis. The inhibitor was also used to study the effects of cathepsin inhibition on macrophage metabolism and phenotype. Due to derailed autophagy and lysosomal signaling, cathepsin inhibition triggered cellular stress and apoptosis of murine macrophages (241) and promoted metabolic changes, which induced the M1 shift in human macrophages, including enhanced glycolysis and synthesis of pro-inflammatory mediators (242).

A specific inhibitor of CatB (CA074) was shown to modulate the immune responses of BALB/c mice infected with *Leishmania major* (243–245). This inhibitor suppressed the Th2 response but augmented the Th1 response, suggesting that CatB functions as an antigen-processing peptidase and preferentially activates the Th2 response. In contrast, treating *L. major*-infected BALB/c mice with CLIK148, a specific inhibitor of CatL, enhances the development of the disease-promoting Th 2 response (246) (**Table 2**).

Furthermore, several reports demonstrated that the selective CatB inhibitor CA-074Me can reduce LMP and cell death in several *in vivo* models, including a mouse model of metastatic cancer (247), a mouse model of Kawasaki disease (248), and during mammary gland involution, where it delays the regression of the gland after weaning (249). The involvement of CatB in the lysosomal cell death pathway and activation of the NLRP3 inflammasome has attracted much attention (250). Recently it was shown that in mouse BMDMs treated with different types of NLRP3 activators (ATP, nigericin or crystals) CatB is necessary for caspase-1 activation, IL-1β production and ASC speck formation (251). CatB presence in the cytoplasm, was shown to be necessary for its interaction with NLRP3 (251). Moreover, CA-074Me was also described to inhibit monosodium urate (MSU)-induced IL-1α and IL-6 production (252). Another cysteine peptidase inhibitor, E64d, reduced srp-6 null *Caenorhabditis elegans-*induced LMP (253).

In addition to cathepsins, their endogenous inhibitors are potential targets for pharmacological inhibition. Silencing of naturally occurring cathepsin inhibitors, such as cystatins and serpins, which attenuate the cytosolic activity of cathepsins and hence LMP-driven apoptosis, may induce apoptosis and consequently be therapeutically useful in autoimmune lymphocyte populations, pathogen-infected cells, and tumor cells. In addition, recent findings (188, 216) suggest that decreasing the intracellular uptake or activation of cystatin F could increase the efficacy of target cell killing by cytotoxic cells (NK cells and CD8+ T cells), thus improving the antitumor immune response.

## Conclusions

Cysteine cathepsins play important roles in maintaining immune cell functions and homeostasis in the immune system. Due to their important roles in pathological conditions such as chronic inflammation, autoimmune disorders, and cancer, cathepsins and their inhibitors represent attractive therapeutic targets. Their emerging roles in the regulation of extra-lysosomal and extracellular proteolysis reveal new targets that can be pharmacologically modulated without disrupting their lysosomal function. To that end, future investigations are needed to study the molecular mechanism(s) controlling lysosomal membrane stability under physiological and pathological conditions as well as to develop new strategies to regulate the activity of cathepsins and their endogenous inhibitors.

## Author Contributions

MPN, UP, and TJ, original draft preparation. MPN, draft editing. JK, review and editing. MPN, visualization. MPN and JK, funding acquisition. All authors contributed to the article and approved the submitted version.

## Funding

This research was funded by Research Agency of the Republic of Slovenia, grant numbers J3-2516 to MPN and P4-0127 to JK.

## Conflict of Interest

The authors declare that the research was conducted in the absence of any commercial or financial relationships that could be construed as a potential conflict of interest.
